# WD-1D-VGG19-FEA: An Efficient Wood Defect Elastic Modulus Predictive Model

**DOI:** 10.3390/s24175572

**Published:** 2024-08-28

**Authors:** Shen Pan, Zhanyuan Chang

**Affiliations:** 1 College of Computer and Control Engineering, Northeast Forestry University, Harbin 150040, China; shenpan866@hotmail.com; 2 College of Information, Mechanical and Electrical Engineering, Shanghai Normal University, Shanghai 200234, China

**Keywords:** near-infrared spectroscopy, 1D-VGG19, finite element analysis, solid wood panel, morphological inversion

## Abstract

As a mature non-destructive testing technology, near-infrared (NIR) spectroscopy can effectively identify and distinguish the structural characteristics of wood. The Wood Defect One-Dimensional Visual Geometry Group 19-Finite Element Analysis (WD-1D-VGG19-FEA) algorithm is used in this study. 1D-VGG19 classifies the near-infrared spectroscopy data to determine the knot area, fiber deviation area, transition area, and net wood area of the solid wood board surface and generates a two-dimensional image of the board surface through inversion. Then, the nonlinear three-dimensional model of wood with defects was established by using the inverse image, and the finite element analysis was carried out to predict the elastic modulus of wood. In the experiment, 270 points were selected from each of the four regions of the wood, totaling 1080 sets of near-infrared data, and the 1D-VGG19 model was used for classification. The results showed that the identification accuracy of the knot area was 95.1%, the fiber deviation area was 92.7%, the transition area was 90.2%, the net wood area was 100%, and the average accuracy was 94.5%. The error range of the elastic modulus prediction of the three-dimensional model established by the VGG19 classification model in the finite element analysis is between 2% and 10%, the root mean square error (RMSE) is about 598. 2, and the coefficient of determination (R2) is 0. 91. This study shows that the combination of the VGG19 algorithm and finite element analysis can accurately describe the nonlinear defect morphology of wood, thus establishing a more accurate prediction model of wood mechanical properties to maximize the use of wood mechanical properties.

## 1. Introduction

Wood is a complex natural composite material, and its unique growth pattern and structural characteristics make it widely used in construction, furniture, and other industrial fields [1,2]. However, growth irregularities and internal defects in wood significantly affect its mechanical properties, especially stiffness and strength. These defects not only reduce the use value of wood but also bring challenges to the processing and application of wood [3,4,5].

To improve the efficiency of wood utilization and the accuracy of performance prediction, researchers have adopted a variety of techniques to study its internal structure and defects. Among them, near-infrared spectroscopy (NIR) has become an important tool to identify and distinguish the internal structure of wood because of its non-destructive and efficient characteristics. [6,7]. Isik et al. combined NIR analysis with a least squares support vector machine to quickly predict wood density and mechanical strength and achieved excellent results [8]. Olsson et al. proposed to measure the fiber angles on the surface of solid wood using laser imaging techniques to predict the status of wood defects and estimate the associated mechanical properties [9,10,11]. Most studies have focused on the mechanical analysis of defect-free samples and defect types, as well as from the perspective of spectral feature optimization and nonlinear modeling. In terms of the board mechanical property test, T Janiak studied the mechanical properties of solid deciduous wood with different moisture contents [12], S Yang confirmed that the mechanical properties of mixed CLT and wood were improved [13], and S Gayda made statistics on the results of the physical and mechanical properties of wood at different ages [14]. SA Huse studied the genetic variation of wood mechanical properties of Eucalyptus clones [15]. These studies show that NIR spectroscopy can provide internal information from a microscopic point of view, which can help identify fiber orientation and defect location. However, the traditional NIR detection method still lacks sufficient description and quantitative analysis of the board morphology and cannot accurately describe the structure and predict the mechanical properties of defective boards. In recent years, deep learning, especially convolutional neural networks (CNNs), has performed well in image classification and pattern recognition [16]. The VGG network is a classic deep convolutional neural network architecture, which can extract multi-level features from images [17]. By combining the one-dimensional convolutional neural network (1D-VGG), NIR spectral data can be effectively classified. Z Wan used VGG for near-infrared spectral classification of Pinus [18], X Xu used near-infrared spectroscopy and GAF-VGG Net to identify the origin of corn seeds [19], and I Işık completed the classification of wheat varieties [20]. AK Sharma used an improved VGG model-based transfer learning method to classify brain tumors [21], C Can used two-dimensional near-infrared images for gesture recognition [22], A Shukla’s application was used in plant phenotyping RGB images for high-resolution near-infrared prediction [23], L Gopinath found a dimensionality reduction method for near-infrared and visible image fusion [24], Y Hong used VGG to fuse NIR and RGB image features to eliminate reflection [25], and Z Dong used a convolutional neural network to classify mango varieties [26]. S Sharma used near-infrared hyperspectral imaging combined with machine learning to evaluate the physical and chemical quality of durian pulp [27], D Mohapatra completed a precision agriculture fruit recognition and grading system based on a deep neural network [28], and SM Hassan used a shallow convolutional neural network for plant disease recognition [29]. Pouyet et al. classified pigments in the short-wave infrared range [30], and Chen et al. completed object recognition modeling for newborn chicks using CNN [31]. Chen et al. used the Resnet50 algorithm to identify the storage time and species of sliced Boletus [32], and DJ A used Resnet and Youjin infrared spectroscopy technology to establish a new classification model for tobacco planting areas [33]. These achievements show that although deep learning, especially VGG, is excellent in image classification and pattern recognition, its application in the field of wood is not yet widespread, and further research and validation are needed. Finite element analysis, which models nonlinear wood by numerical approximations, has been applied mostly to the effects of heterogeneity in wood material distribution under a variety of stress and strain fields [34,35]. Hu et al. proposed a microstructure-based multi-surface failure criterion to describe the brittle and ductile failure mechanisms of wood, but they described the defect as a cone and did not divide the wood regions [36]. Christoph Hackspiel divided the wood into four regions according to the fiber trend of wood: knot region, fiber deviation region, transition region, and net wood region, and simulated the stress of double-knot wood by the finite element method. However, they did not accurately define the transition zone and the fiber deviation zone [37]. Kiraly T modeled the growth ring of Norway spruce wood beams in the finite element calculation [38], and Sobczak-Pi Piąstka J simulated glulam beams in the case of oblique bending [39]. However, most of the existing finite element analyses of wood morphology and mechanical properties involve the construction of homogeneous material regions or simple morphological descriptions, failing to accurately define regions and analyze internal structural changes and mechanical mechanisms.

This study aims to propose a new method for wood defect detection and mechanical properties prediction by combining near-infrared spectroscopy, the 1D-VGG deep learning algorithm, and finite element analysis. In this study, the region definition theory proposed by Christoph [24] was used, the VGG19 feature optimization method was used to complete the effective feature extraction, the four regions of the defective solid wood panel were identified and classified, and the surface two-dimensional image was inverted, and then a more accurate three-dimensional model describing the solid wood panel structure was established, and the elastic modulus was predicted by the finite element analysis software. The innovation lies in the combination of deep learning with traditional NIR spectroscopy and finite element analysis, which not only improves the accuracy of wood internal defect detection but also enhances the accuracy of mechanical property prediction. This method is not only of great significance in theory, but also provides a new idea for the efficient use of wood in actual production, avoiding the waste of wood properties and contributing to the reduction of carbon emissions.

## 2. Materials and Methods

### 2.1. Near-Infrared Spectroscopy Data Acquisition

Larch sample boards with a size of 200 × 50 × 20 mm were used in the experiment and were conditioned to an equilibrium moisture content at 65% relative humidity and 20 °C before all tests. The spectral acquisition equipment was a Finnish SPECIM industrial hyperspectral camera FX series, with a wavelength range of 935.61~1720.23 nm, a spectral resolution of 3.45~3.58 nm, and a total of 224 bands. The near-infrared spectrum camera carried out linear array push-broom type data acquisition on the larch wood defect board sample, so that the board surface image was in one-to-one correspondence with the spectrum data by taking a pixel point as a unit. According to that plate image, the corresponding spectral data were exported and stored in different areas.

**Figure 1:**
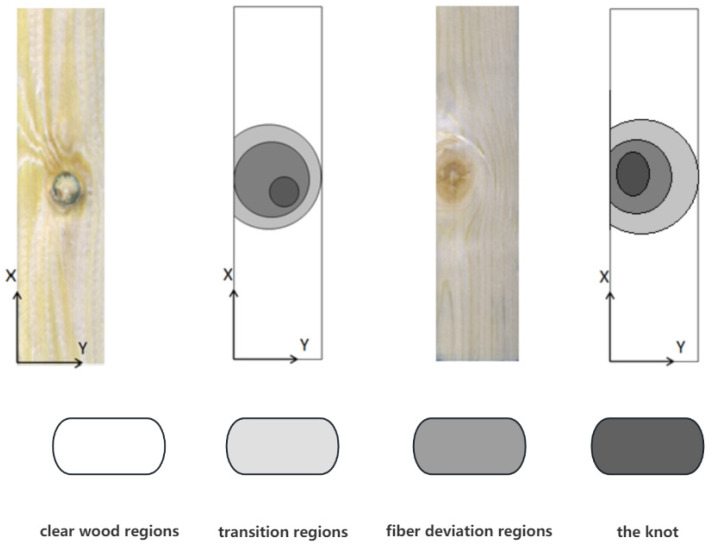
Schematic diagram of the division into four areas of the larch plate specimen.

The sample and area schematics are shown in Figure 1. According to the research of Hu and Christoph Hackspiel [37,38], the fiber growth direction on the plate gradually changes from parallel to the plate surface to surrounding defects at an angle with the plate surface. According to the fiber trend of the board, the area where the fiber growth direction is parallel to the *X* axis is defined as the net wood area, the area where the fiber growth direction is parallel to the *XY* plane but has an angle with the *X* axis is defined as the transition area, the area where the fiber growth direction has an angle with the *XY* plane is defined as the fiber deviation area, and the defect is defined as the knot area.

**Figure 2:**
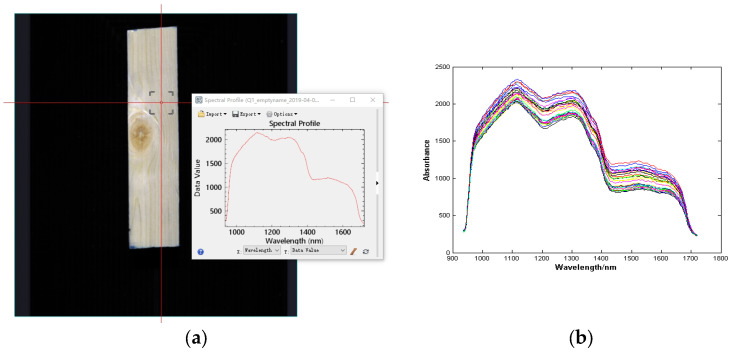
Schematic diagram of spectrum acquisition (**a**) Spectrum acquisition interface for ENVI5.3; (**b**) NIR spectral image.

Near-infrared spectra were collected using ENVI 5.3 by randomly selecting points from four areas divided by seven plates, where 270 points were randomly collected in each area. Then, 189 points were randomly selected from each region as the training set, the remaining 162 points as the validation set, and 162 points as the test set. Subsequently, another 19 plates were selected for surface morphology inversion. See Figure 2a for the collection interface of regional feature points and see Figure 2b for the spot spectral image.

### 2.2. D-VGG19 and 3D Finite Element Modeling

**Figure 3:**
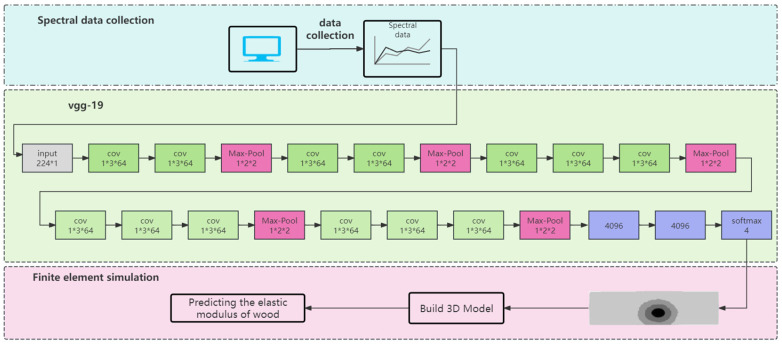
1D-VGG19-FEA algorithm is used to identify the knot area of larch board and predict the elastic modulus process.

The board can be freely combined into different structural forms according to the four wood regions, and the mechanical properties of the same size board vary greatly due to different structures. Based on the front-end numerical analysis model of VGG19 algorithm, define and invert the shape of larch defective plate, construct the three-dimensional model of multi-region defective structure, and predict the elastic modulus by finite element analysis, as shown in Figure 3.

Before inputting the data into the VGG19 model, the near-infrared data were normalized to meet the input requirements of the VGG19 model. The standard normal transformation method is used here. Liu’s research proves that the standard normal transformation is an effective method for processing near-infrared spectral data [40]. In this experiment, we used the VGG19 architecture to classify the near-infrared spectral data in 224 bands. VGG19 is a deep convolutional neural network, including 16 convolutional layers and 3 fully connected layers, with 19 weight layers. When dealing with NIR data, we cannot directly use the two-dimensional convolution kernel (such as 3 × 3) because the data is one-dimensional. Therefore, the convolution kernel in the VGG19 architecture should be adjusted to a one-dimensional convolution kernel (1 × 3). The following describes the experimental process in detail, including the settings of the input layer, the convolution layer, and the pooling layer.

1. Input layer:

Input shape: since the NIR spectral data have 224 wavelength points, we set the shape of the input data to be (224, 1), where 224 is the number of wavelength points and 1 represents the characteristics of each wavelength point.

2. Convolutional layer:

Convolution kernel size: a 1 × 3 convolution kernel is used because the data are one-dimensional, so a one-dimensional convolution kernel is used for processing. Number of convolution kernels: as the network is deepened, the number of convolution kernels is gradually increased to capture more features. In the one-dimensional VGG19, the number of convolution kernels is gradually increased from 64 to 512. Activation function: the ReLU (Rectified Linear Unit) activation function is used after each convolutional layer to increase the nonlinear capability of the network. Specific convolution layer settings are shown in Table 1:

3. Pool layer

Pooling layer configuration: each convolution block is followed by a 1 × 2 maximum pooling layers, which is used to down sample and reduce the size and computation of the feature map while preserving the main features. The step of the pooling layer is 2.

4. Fully connected layer

After going through multiple layers of convolution and pooling, the feature map is flattened into a vector and fed into the fully connected layer. The first fully connected layer has 4096 neurons and uses the ReLU activation function. The second fully connected layer has 4096 neurons and uses the ReLU activation function.

5. Output layer:

Using the SoftMax activation function, the number of output nodes is 4, corresponding to four classification classes.

By adjusting the convolution kernel in the VGG19 architecture to a one-dimensional convolution kernel (1 × 3), we can effectively process the near-infrared spectral data at 224 wavelength points. The specific setup includes the use of multiple 1 × 3 convolutional layers followed by a 1 × 2 max-pooling layer in each convolutional block, and the use of fully connected layers for the final classification. Through this adjustment, the VGG19 architecture can adapt to the processing requirements of one-dimensional data, and effectively classify the near-infrared spectral data into four categories.

## 3. Experiments and Analysis

In this experiment, we used the 1D-VGG19 framework to classify the NIR spectral data of 224 bands. VGG19 is a deep convolutional neural network, including 16 convolutional layers and 3 fully connected layers, with a total of 19 weight layers. The wavelength points of the near-infrared spectral data need to be normalized to scale the data to between 0 and 1. This can ensure that the input data are suitable for the processing of the neural network and improving the training effect and stability of the model. The normalized data are divided into a training set, validation set, and test set for model training and performance evaluation. The division ratio is 752 for training, 164 for validation, and 164 for testing.

The results are shown in Table 2. When the number of iterations is small, the model training is insufficient, and the classification effect is poor. With the increase in the number of iterations, the classification accuracy increases. When the number of iterations is large enough, the discrimination results of the model tend to be stable, and the model reaches the convergence state. The number of iterations chosen for this study is 1000. Table 3 shows the comparison of the proposed model with other classification algorithms. It can be seen from the table that the proposed 1D-VGG19 model has higher overall accuracy than other classification algorithms.

Choosing an appropriate model requires a trade-off between accuracy, computational cost, and implementation difficulty. VGG19 has a 19-layer deep network, which can extract more features and is suitable for complex data. Because of the large number of layers, the calculation is large, the training time is long, and the hardware requirements are high. In addition, there are many network parameters, which may lead to over-fitting and require a large amount of data for training. DNN can design various structures to accommodate different types of data and tasks. Its network structure is relatively simple and easy to implement and debug. However, for complex data, DNN has difficulty capturing fine-grained features and does not perform as well as deep convolutional neural networks. ResNet50 has a network of 50 layers deep and is able to extract more complex features. It solves the problem of gradient vanishing in deep network training through residual blocks, which makes the training more stable. However, similar to VGG19, ResNet50 has a large amount of computation and parameters, a long training time, and high hardware requirements. Its structure is more complex than VGG, and it is more difficult to debug and optimize. VGG16 has a 16-layer deep network, which can extract more features. Although it is simpler than ResNet50, it can still achieve higher performance and is easy to implement and debug. Although the number of layers is less than VGG19, the amount of calculation is still large, and the training time is long. There are many network parameters, which may lead to over-fitting and require a large amount of data for training. VGG19 is the best choice when high precision is required and hardware conditions permit; VGG16 is a better choice when computing resources are limited but high performance is still required; ResNet50 is a good choice when deep network training problems need to be solved and there are enough computing resources; DNN provides a high degree of flexibility for rapid prototyping and debugging.

**Figure 4:**
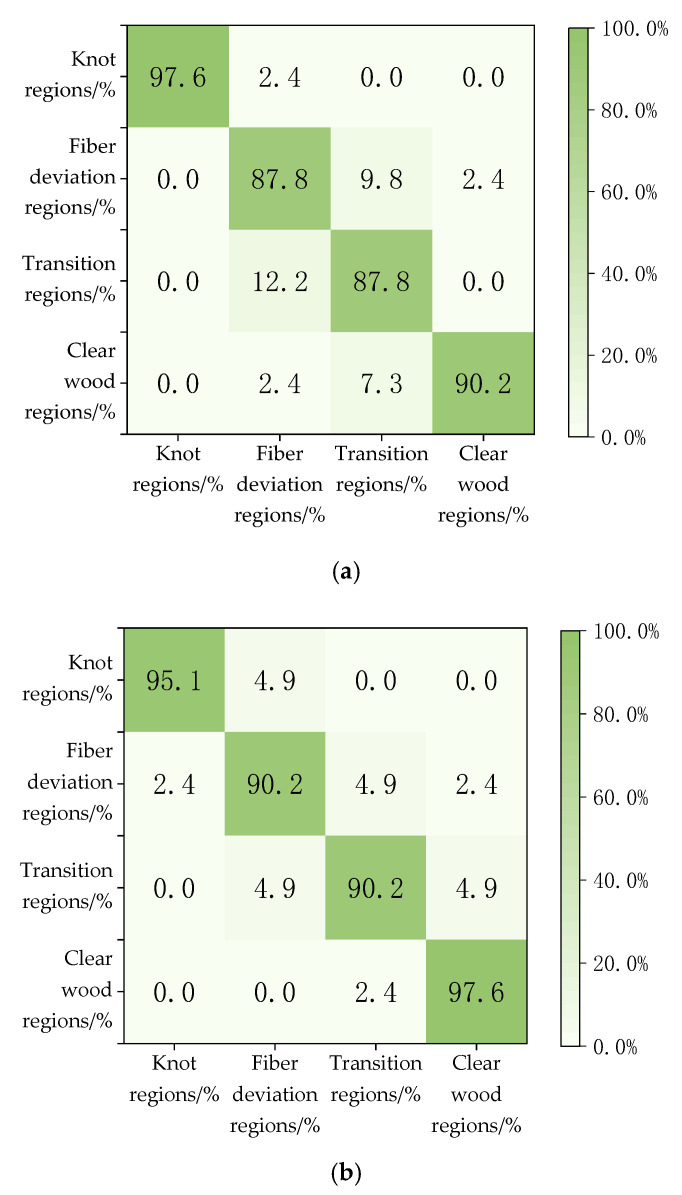

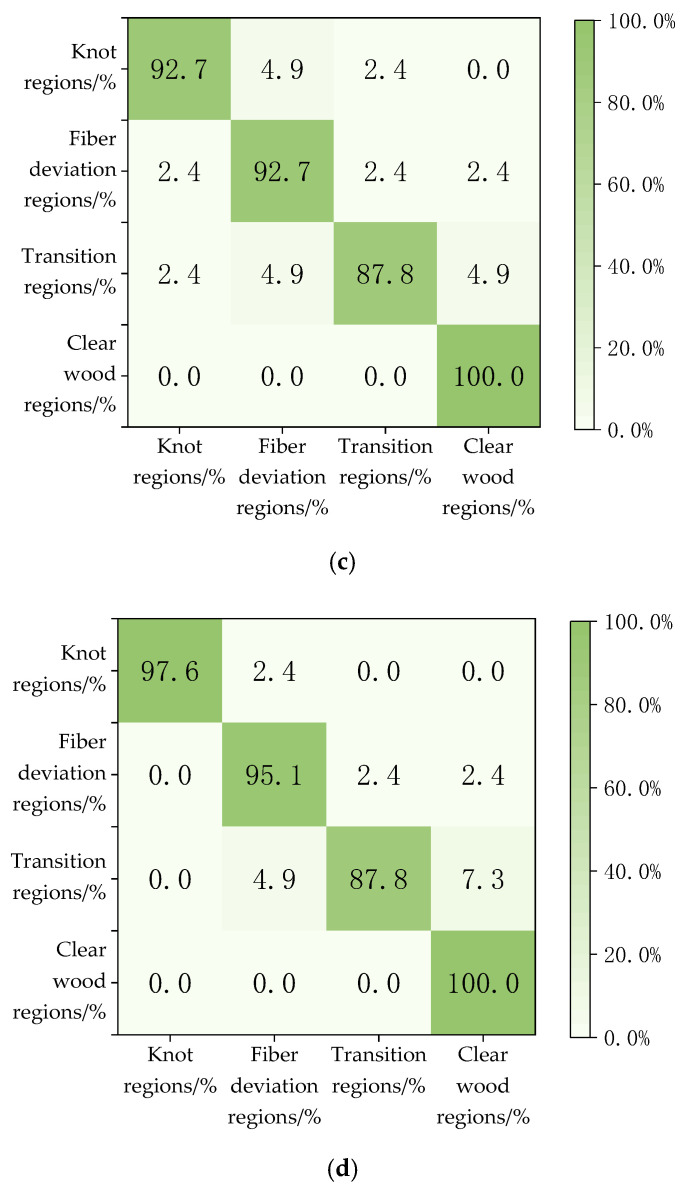
Confusion matrix of different model classification methods (**a**) DNN; (**b**) ResNet50; (**c**) VGG16; (**d**) VGG19.

Figure 4 presents the confusion matrices for different classification methods, and Table 3 lists the average accuracy of these methods. From these data, it can be observed that all four classification methods perform well in distinguishing between the clear wood zone and defect zone. However, in the classification of the transition zone and fiber deviation zone, VGG16 and VGG19 show significantly better results. Overall, VGG19 demonstrates the best performance among all the methods, offering the most optimal comprehensive performance.

The results in Table 4 show that using the VGG19 classification method, the identification accuracy of the knot area is 95.1%, the fiber deviation area is 92.7%, the transition area is 90.2%, the net wood area is 100%, and the average accuracy is 94.5%. The VGG19 algorithm can effectively classify the transition zone, fiber deviation zone, and net wood zone of wood, but the accuracy of the transition zone is slightly lower. Understanding irregular growth defects has a positive effect on the study of wood stiffness and strength, which can help to bring economic benefits to higher grade and higher yield wood producers.

## 4. Results and Discussion

### 4.1. Nonlinear Sheet Shape Inversion

**Figure 5:**
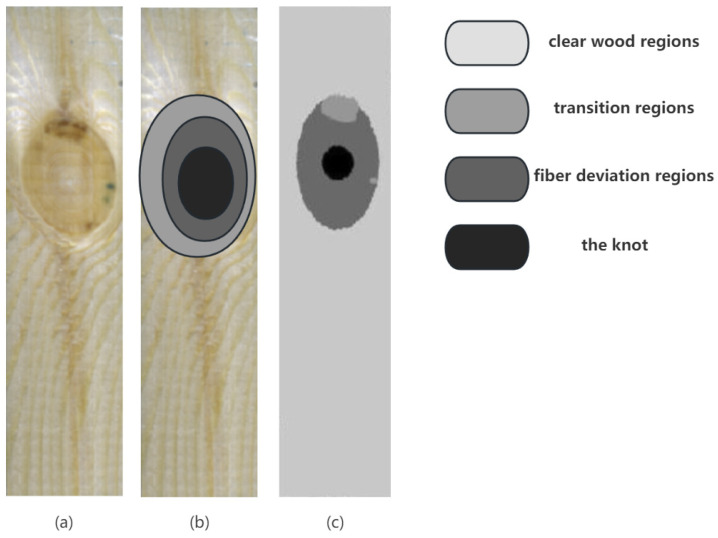
Planar form of wood to counter the intent of demonstration. (**a**) Sample; (**b**) Linear Model; (**c**) Comparison of VGG19 modeling effect.

**Figure 6:**
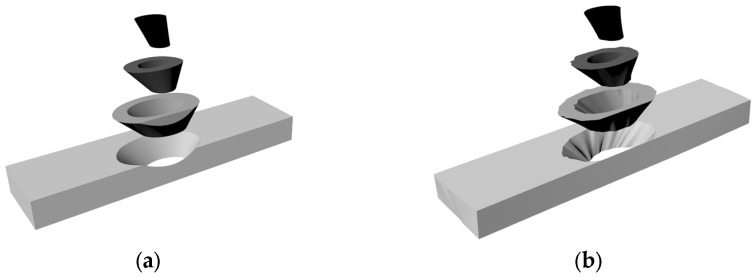
Wood three-dimensional shape anti-demonstration intention. (**a**) Linear Model; (**b**) Nonlinear Model.

**Figure 7:**
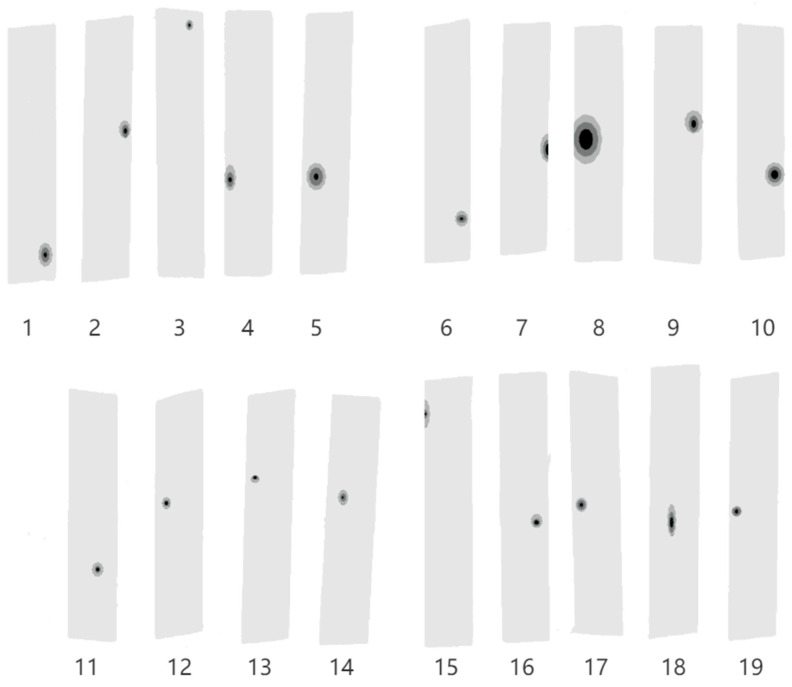
Inversion effect map of 19 Specimens from No.1 to No.19.

The lack of an accurate description of the morphology of plates containing defects makes it difficult to study these defects through physics-based numerical simulations. The linear model has room for improvement in the prediction of mechanical properties of sheet metal. Near-infrared spectral information is used to describe the wood surface, and the comparison chart of wood surface morphology obtained by nonlinear inversion is shown in Figure 5. Figure 6 shows the internal structure of the linear model (6-a) and the nonlinear model (6-b), and Figure 7 is the inversion diagram of the 19 specimens in this test.

Because of the high anisotropy of wood, the influence of fiber orientation on the mechanical properties of wood is very significant. The stiffness and strength of wood on a dimensional scale are highly variable and are mainly caused by fiber deviations in the vicinity of defects. The lack of information on the effects of growth irregularities on the mechanical properties of wood panels prompted us to investigate these defects through experimental measurements and physics-based numerical simulations. Wood is a nonlinear material, and the morphology of defects cannot be simply replaced by a regular cone. The density of the transition zone and the fiber deviation zone is also different from that of the net wood, which leads to the deviation of their properties. Wood has complex morphological changes between the knot area and the clean wood area, so it is necessary to accurately define the shape and boundary of the four areas of wood, establish the nonlinear description model of each area on the surface of the board, and classify the wood to maximize its utilization.

### 4.2. Establishment of Finite Element Analysis Model and Prediction of Elastic Modulus of Larch Lumber

Wood is a complex natural fiber composite. From the macroscopic structure, wood can be seen as a composite of materials with different properties, and there are multi-level composite effects. Therefore, composite mechanics should be the main tool to study the macroscopic mechanical properties of wood. It is necessary to study the distribution and transfer theory of macro-external forces in different areas of the board, that is, to study the stress–strain relationship of macro-tissue in different structures, and to verify it through the compressive strength test of wood parallel to grain.

**Figure 8:**
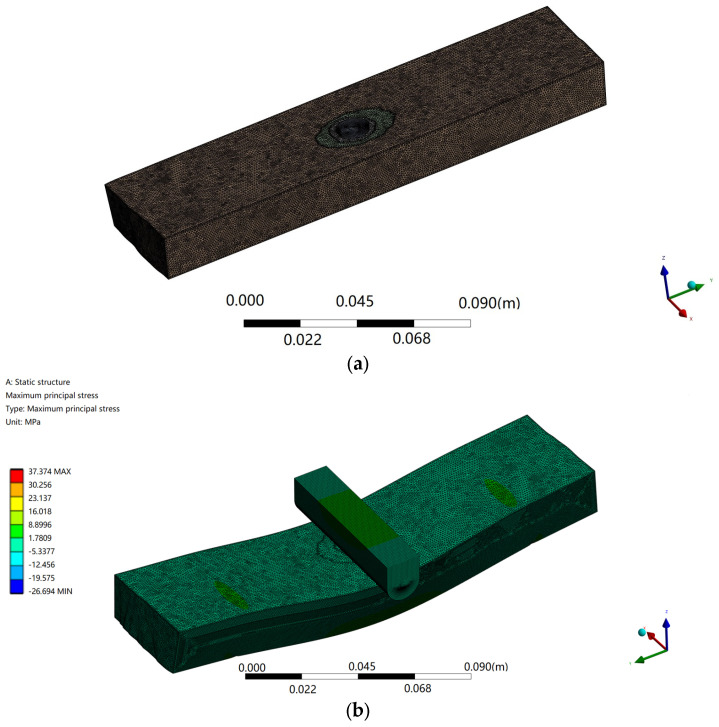

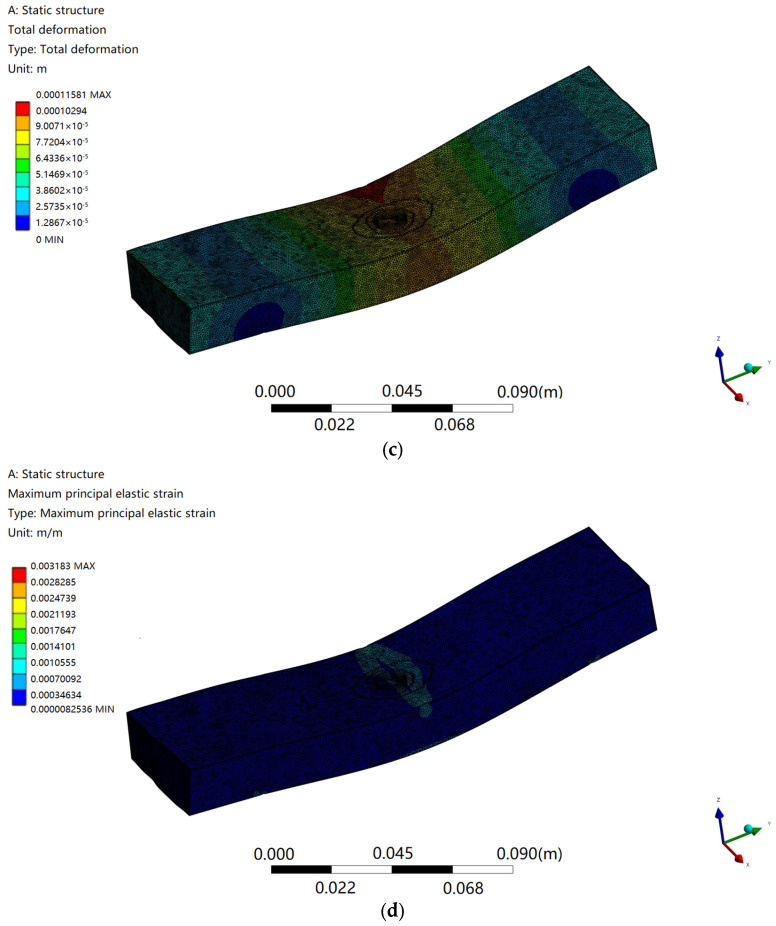
Finite element solution process (**a**) Divide the grid; (**b**) Set Solve Control Option; (**c**) Comparison of VGG19 modeling effect; (**d**) Maximum elastic strain.

UG 3D-modeling software was used to build a 3D model according to the inverted figure of the specimen, and then imported into Ansys Workbench through the common interface. The physical properties of the model are determined by the three parameters of Young’s elastic modulus, Poisson’s ratio, and material density, and the finite element model is created by meshing, as shown in Figure 8a. Constraints, including boundary and initial conditions, are applied, fixed supports are applied on both sides of the material, loads are applied, and solution control options are set as shown in Figure 8b. After you optimize the meta-analysis, you can view, analyze, and verify the results of the solution. The calculation results can be displayed by color nephogram and contour map, and the total deformation and maximum elastic strain are shown in Figure 8c,d. Table 5 is a comparison of the prediction values of different methods, and Table 6 is a comparison of the evaluation indicators of the prediction values of different methods.

The results in Table 4 are the test set experimental results. The results showed that the identification accuracy of the knot area was 95.1%, the fiber deviation area was 92.7%, the transition area was 90.2%, the net wood area was 100%, and the average accuracy was 94.5%. The Vgg19 prediction model uses prediction group data to predict the error range between 2% and 10%, with an RMSE of about 598.2 and an R2 of 0.91. The RMSE of the VGG16 prediction model is about 801.5 with an R2 of 0.83. The RMSE of the RESNET50 prediction model is about 868.1 with an R2 of 0.81. The RMSE of the DNN prediction model is about 991.2, and the R2 is 0.75. The linear model predicts that the RMSE of the model is about 1124 and the R2 is 0.67. The VGG19 model performed best in this prediction task with a small error range, the lowest RMSE, and the highest R2, indicating that it has the strongest prediction ability and the best data fitting effect. VGG16 model and ResNet50 model have better prediction performance but are slightly inferior to VGG19. DNN and linear models perform significantly worse than deep learning models, especially linear models, which have the highest RMSE, the lowest R2, and the worst predictive ability. Overall, the VGG19 model exhibits the best performance in this task and is suitable as the preferred prediction model.

## 5. Conclusions

It can be seen from the results that the linear model cannot accurately describe the morphology of the wood area and underestimates the bearing capacity of the wood. In contrast, the combination of near-infrared spectral features with the one-dimensional VGG19 and finite element analysis (FEA) algorithm allows for more accurate classifications of wood regions and more accurate nonlinear morphological inversion and performance predictions. In this paper, the idea of heterogeneous material is used to model the larch specimen, and the wood is idealized as a heterogeneous structural material with four regions. The four regions of the plate were modeled by VGG19 near-infrared spectroscopy, the nonlinear morphology of the plate surface was inversed, the nonlinear three-dimensional model was established, and the elastic modulus was predicted. The test results show that the one-dimensional convolution kernel of VGG19 can effectively process the near-infrared spectral data of 224 wavelength points, and quickly identify the wood region with the highest accuracy after 1000 iterations. VGG19 reduces the redundancy between features and shortens the classification time. The prediction results of the three-dimensional model established by the inversion diagram are more accurate than those of the linear conical defect model and other prediction methods, and the problem of inaccurate descriptions of the plate morphology is optimized. The method effectively improves the utilization rate of the performance of the plate and is beneficial to realizing carbon neutralization and reducing carbon emission. In the experiment, the images of plates with defects are selected, and the practicability of the algorithm is improved by combining the complex and diverse mechanical properties prediction research. The next step is to focus on how to reduce the time required to calculate the convex optimization problem and improve the efficiency of operation to meet the requirements of online sorting of mechanical properties of sheet metal.

## Figures and Tables

**Table 1:** Convolution layer settings of VGG19.

	Number of Roll Base Layers	Number of Convolution Kernels	Convolution Kernel Size	Stride	Fill
blook1	2	64	1 × 3	1	1
blook2	2	128	1 × 3	1	1
blook3	2	256	1 × 3	1	1
blook4	2	512	1 × 3	1	1
blook5	2	512	1 × 3	1	1

**Table 2:** Comparison of classification of training set of VGG19.

Number of Iterations	Accurate Classification Quantity	Training Set Accuracy/%
300	495	65.8
500	578	76.9
800	658	87.5
1000	707	94.2
2000	701	93.2

**Table 3:** Comparison of the average accuracy of wood classification.

Method	DNN/%	Resnet50/%	VGG16/%	VGG19/%
Correct quantity	149	153	153	156
Average accuracy	90.9	93.3	93.3	95.1

**Table 4:** Comparison of classification accuracy of training sets for wood.

	Knot Regions/%	Fiber Deviation Regions/%	Transition Regions/%	Clear Wood Regions/%	Average Accuracy/%
Test set classification accuracy/%	95.1	92.7	90.2	100	94.5
Verification set classification accuracy/%	97.6	95.1	87.8	100	95.1

**Table 5:** Comparison of prediction results of elastic modulus.

No of the Plate	Raw Data (MPa)	VGG19-FEA (MPa)	VGG16-FEA (MPa)	Resnet50-FEA (MPa)	Dnn-FEA (MPa)	Linear Model (MPa)
1	10,280.8	10,070.1	9661.8	11,278.2	9781.5	10,720.9
2	12,605.0	12,947.9	11,668.3	12,371.5	11,756.3	13,070.2
3	14,740.8	15,376.5	13,576.2	13,980.7	15,809.2	15,582.0
4	11,384.7	11,067.7	12,149.5	10,542.6	12,819.3	11,921.6
5	15,777.1	17,317.2	16,664.9	14,528.1	16,364.5	17,273.6
6	15,210.0	16,343.3	14,431.3	13,982.7	13,605.3	16,987.2
7	14,913.5	15,416.8	15,557.9	15,954.1	16,058.9	16,465.2
8	11,939.8	11,713.5	12,697.2	12,471.1	12,420.1	12,322.0
9	12,601.4	12,009.9	11,659.6	13,710.1	12,297.3	13,384.2
10	14,207.0	14,273.3	13,310.7	14,2980.	13,191.7	15,481.8
11	12,444.00	12,046.1	13,031.6	11,825.2	13,258.9	12,791.9
12	13,873.5	13,549.9	14,771.9	14,775.5	12,920.0	15,569.2
13	9443.2	9628.1	9907.1	9097.9	8756.3	10,117.6
14	14,699.8	15,260.8	14,033.5	15,627.2	15,156.6	15,962.7
15	11,188.1	11,347.1	11,765.7	12,319.2	10,671.9	11,651.8
16	14,692.6	13,714.9	15,733.1	13,578.1	15,083.7	15,799.5
17	15,951.6	16,092.7	15,204.9	16,725.8	17,831.1	17,947.1
18	10,822.5	10,406.7	11,556.2	11,821.3	9782.2	11,244.2
19	10,669.1	10,493.7	11,448.1	10,532.1	11,942.8	12,003.6

**Table 6:** Comparison of evaluation indicators of prediction results.

Evaluation Indicators	VGG19-FEA	VGG16-FEA	Resnet50-FEA	Dnn-FEA	Linear Model
MSE	598.2	801.5	868.1	991.2	1124
R2	0.91	0.83	0.81	0.75	0.67

## Data Availability

The original contributions presented in the study are included in the article, further inquiries can be directed to the corresponding author.
